# Correlation of Serum Uric Acid Levels with Nonculprit Plaque Instability in Patients with Acute Coronary Syndromes: A 3-Vessel Optical Coherence Tomography Study

**DOI:** 10.1155/2018/7919165

**Published:** 2018-02-07

**Authors:** Donghui Zhang, Ruoxi Zhang, Ning Wang, Lin Lin, Bo Yu

**Affiliations:** Department of Cardiology, Key Laboratories of Education Ministry for Myocardial Ischemia Mechanism and Treatment, 2nd Affiliated Hospital of Harbin Medical University, Harbin 150086, China

## Abstract

Elevated serum uric acid (SUA) level is known to be a prognostic factor in patients with acute coronary syndrome (ACS). However, the correlation between SUA level and coronary plaque instability has not been fully evaluated. The aim of this study was to investigate the association between SUA level and plaque instability of nonculprit lesions in patients with ACS using optical coherence tomography. A total of 150 patients with ACS who underwent 3-vessel optical coherence tomography were selected. Patients were classified into 3 groups according to tertiles of SUA level. There was a trend towards a thinner fibrous cap (0.15 ± 0.06 versus 0.07 ± 0.01 versus 0.04 ± 0.01 mm^2^, *p* < 0.001) and a wider mean lipid arc (169.41 ± 33.16 versus 177.22 ± 37.76 versus 222.43 ± 47.65°, *p* < 0.001) with increasing SUA tertile. The plaques of the high and intermediate tertile groups had a smaller minimum lumen area than the low tertile group (6.02 ± 1.11 versus 5.38 ± 1.28 mm^2^, *p* < 0.001). In addition, thin-cap fibroatheromas, microvessels, macrophages, and cholesterol crystals were more frequent in the high tertile group than the low and intermediate groups. Multivariate analysis showed SUA level to be a predictor of plaque instability.

## 1. Introduction

Several epidemiological studies showed that increased levels of serum uric acid (SUA), the main end product of purine metabolism, are associated with cardiovascular diseases [1]. Elevated levels of uric acid are independent predictors of 1-year mortality across the whole spectrum of patients with acute coronary syndromes (ACSs), treated with percutaneous coronary intervention [2]. It has been found that increased SUA is associated with endothelial dysfunction [3], antiproliferative effects, high oxidative stress, generation of free radicals, and thrombus formation, all promoting atherosclerosis and its sequelae. Although the possibility of an association between elevated SUA level and cardiovascular disease has been recognized for 130 years [4, 5], the role of SUA levels as a risk factor or a risk marker for nonculprit plaque instability remains debated. The effect of hyperuricemia on coronary atherosclerosis, evaluated using intravascular ultrasound (IVUS) or coronary computed tomographic angiography, has been reported, and it has been stated that SUA levels are associated with coronary plaque components on IVUS [6–8]. There is no clinical study investigating the effects of SUA levels on nonculprit plaque instability by optical coherence tomography (OCT). Intracoronary OCT is an emerging technology that enables cross-sectional vascular imaging with approximately 10–20 *μ*m resolution. The relatively high resolution of OCT makes it a powerful tool for both quantitative plaque analysis and qualitative plaque characterization [9, 10]. OCT can provide detailed in vivo information on atherosclerotic plaques inside vessels, including tissue characteristics and fibrous-cap thickness. Thus, the objective of our study was to investigate the correlation between SUA level and the stability of nonculprit plaques in patients with ACS.

## 2. Methods

### 2.1. Ethics Statement

The present study was approved by the Research Ethics Committee of the Second Affiliated Hospital of Harbin Medical University, China. Data analysis was blinded to the patients' identification information; therefore, no informed consent was required.

### 2.2. Patients and Study Design

We identified 150 patients admitted with the diagnosis of ACS who underwent 3-vessel OCT examinations at the 2nd Affiliated Hospital of Harbin Medical University (Harbin, China) between August 2007 and December 2010. All nonculprit or nontarget lesions with >30% diameter stenosis on coronary angiogram were analyzed. The major criteria for exclusion were cardiogenic shock, congestive heart failure, chronic total occlusion, left main coronary artery disease, renal failure, antihyperuricemic agent use before admission, hemodialysis, no stent implantation, emergent surgery or mechanical support (e.g., extracorporeal membrane oxygenation), cardiopulmonary arrest, in-hospital adverse events, and a target lesion in the stented segment. Patients who had no analyzable nonculprit plaque (plaque burden > 20% and at least 5 mm length) were also excluded. Four patients were excluded due to poor image quality. Included patients were classified into 3 groups according to tertiles of SUA level.

ACS included ST-elevation-myocardial infarction (STEMI), non-STEMI, and unstable angina (UA). STEMI was defined as continuous chest pain >30 min, ST-segment elevation > 0.1 mV in ≥2 contiguous leads or new left bundle-branch block on electrocardiogram, and elevated cardiac markers. Non-STEMI was defined as ischemic symptoms with elevated cardiac markers (troponin T/I or creatine kinase-MB) but without ST-segment elevation on 12-lead electrocardiogram. Unstable angina was defined as rest, new-onset, or accelerating angina. Nonculprit lesions were defined as plaques viewed on an angiogram that had not been treated. Each plaque was separated by at least 5 mm from the edge of any other plaque or implanted stent edge.

### 2.3. OCT Images and Analysis

Images were acquired using a commercially available frequency domain (C7­XR OCT Intravascular Imaging System, St. Jude Medical, St. Paul, MN, USA) or time domain (M2/M3 Cardiology Imaging System, Light Lab Imaging, Inc., Westford, MA, USA) OCT system. The intracoronary OCT imaging technique has been described previously [11]. In brief, in the frequency domain OCT system, a 2.7 F OCT imaging catheter (Dragonfly, Light Lab Imaging, Inc.) is advanced distal to the lesion, and automated pullback is initiated in concordance with blood clearance by the injection of contrast media or dextran. In the time domain OCT system, an occlusion balloon (Helios, Light Lab Imaging, Inc.) is inflated proximal to the lesion at 0.4 to 0.6 atm during image acquisition. The imaging wire is automatically pulled back from a distal to a proximal position at a rate of 1.0 to 3.0 mm/s, and saline is continuously infused from the tip of the occlusion balloon. Any discordance between the two independent reviewers was resolved by consensus.

**Figure 1:**
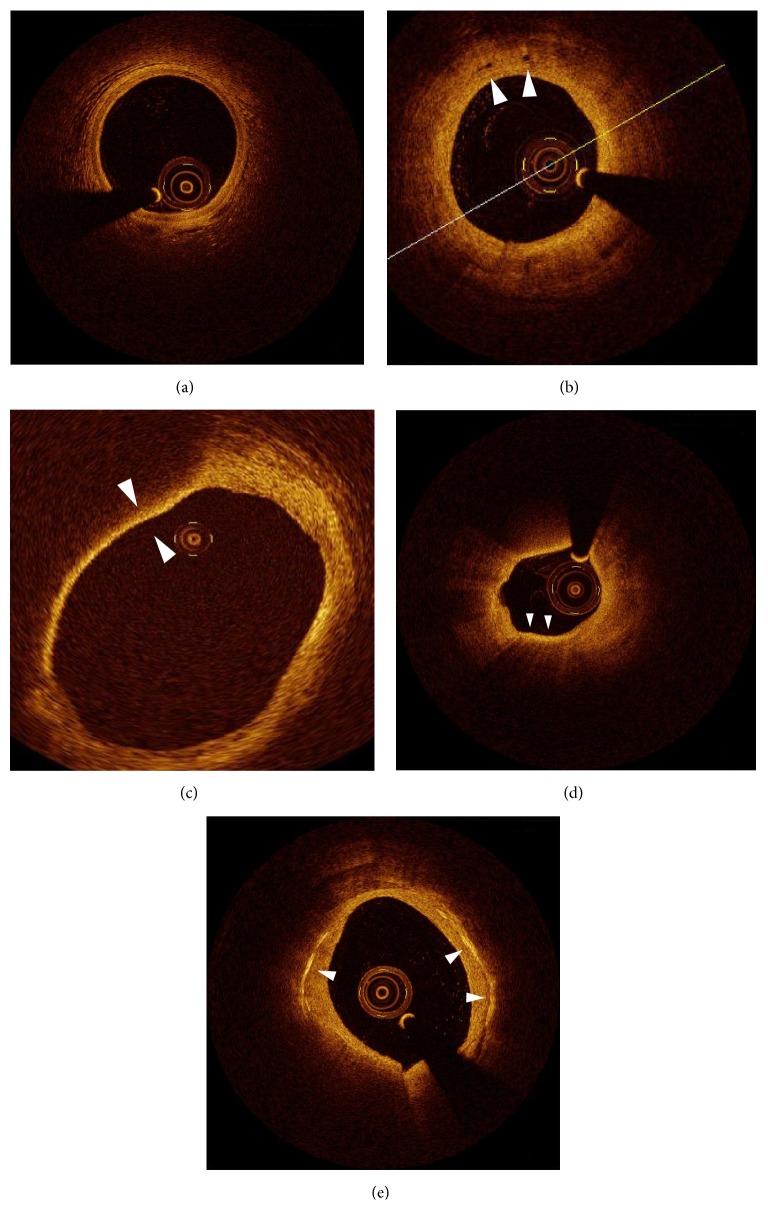
OCT findings related to atherosclerosis included a determination of the presence of lipids and microstructure in the plaque. (a) Normal vessel with 3 layers. (b) Microvessels (white arrow). (c) TCFA (cap thickness, <65 *μ*m). (d) Macrophages are identified as a bright spot region in the context of thin-cap fibroatheroma. (e) Cholesterol crystal (white arrow).

**Figure 2:**
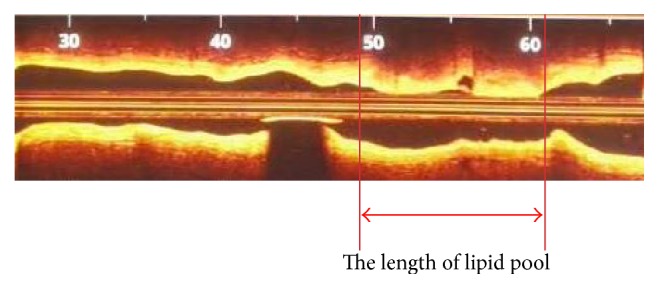
Longitudinal extent of lipid pool assessed by optical coherence tomography (OCT). The length of lipid pool was measured as the consecutive longitudinal length of lipid pool at culprit plaque assessed by OCT.

Assessment of atherosclerosis included a determination of the presence of lipids and microstructure in the plaque (Figure 1). Fibrous plaques were identified as homogenous signal-rich areas. Lipid plaques were defined as signal-poor regions with diffuse borders [10, 12, 13]. When lipid was present in ≥90° in any of the cross-sectional images within the plaque, it was considered to be a lipid-rich plaque [13–16]. In lipid-rich plaques, lipid arc was measured at every 1 mm interval throughout the entire length of each lesion and the mean of the values was determined. Lipid length was also measured on longitudinal view. Lipid volume index was defined as the averaged lipid arc multiplied by lipid length. Fibrous-cap thickness of the lipid plaque was measured three times at its thinnest part, and the values were averaged [11]. Thin-cap fibroatheroma (TCFA) was defined as a plaque with >90° maximum lipid arc and cap thickness < 65 *μ*m. Macrophage accumulation was defined as increased signal intensity within a lesion, accompanied by high signal attenuation casting a heterogeneous shadow [17]. A microvessel was defined as a sharply delineated signal-poor void with a diameter of 50–300 *μ*m, which was not connected to the vessel lumen and was noted on >3 consecutive frames [18]. Cholesterol crystals were defined as thin, linear regions of high intensity within the plaque [19]. The length of lipid pool was measured as consecutive longitudinal length of lipid pool at culprit plaque assessed by OCT (Figure 2), as reported previously [20].

### 2.4. Statistical Analysis

Quantitative variables are expressed as mean value ± standard deviation, and qualitative variables are expressed as total number and percentage. The independent two-sample *t*-test or one-way analysis of variance (ANOVA) with post hoc Student-Newmen-Keuls test was used to assess the differences between multiple sets of data. Categorical variables were also compared using the chi-square or Fisher's exact test. Univariate and multivariate logistic regression analyses were used to identify independent predictors of plaque instability. Statistical significance was indicated when a two-sided *p* value was <0.05. All statistical analyses were performed using SPSS version 19.0 (SPSS Inc., Chicago, IL, USA).

## 3. Results

### 3.1. Clinical Characteristics

Tertiles of SUA level were as follows: low tertile, <258 *μ*mol/L; intermediate tertile, 258 to 375 *μ*mol/L; and high tertile, >375 *μ*mol/L. The clinical characteristics of the three groups are summarized in Table 1. There were no significant differences in the prevalence of age, hypertension, diabetes mellitus, dyslipidemia, and current smoking among the three groups. There were also no significant differences in the levels of creatinine, low density lipoprotein, high density lipoprotein, TG, and TC. However, there was a significant difference in the level of high-sensitivity C-reactive protein. The prevalence of males was higher in the intermediate tertile group than in the low tertile group.

### 3.2. OCT Findings

Plaque-based comparison of OCT findings in the groups with different SUA levels is shown in Table 2. There were no significant differences in plaque length and lipid length among the three groups. Compared with plaques of patients with different SUA levels, those of patients with higher SUA levels had a thinner fibrous cap and a wider mean lipid arc. The plaques of the high and intermediate tertile groups had a smaller minimum lumen area than the low tertile group. In addition, TCFA, microvessels, macrophage, and cholesterol crystals were more frequent in the high tertile group than the low and intermediate tertile groups.

Correlations between OCT findings and SUA levels are shown in Table 3. There was no correlation of plaque length and lipid length with SUA levels. On the other hand, SUA levels correlated significantly with mean lipid arc and inversely correlated with minimum lumen area and fibrous-cap thickness.

In univariate and multivariate regression analyses, SUA was significantly associated with plaque instability (Table 4).

## 4. Discussion

To the best of our knowledge, this is the first study investigating the association between SUA level and coronary plaque characteristics in nonculprit lesions in patients with ACS using 3-vessel OCT imaging. OCT provided accurate measurements of coronary lumen with excellent intraobserver reproducibility. Otherwise, OCT is able to clearly differentiate plaque components such as intimal hyperplasia, lipid, or calcium accumulation in coronary arteries and also visualize fine structural changes in coronary arterial walls. The present study showed that SUA levels correlated significantly with mean lipid arc and inversely correlated with minimum lumen area and fibrous-cap thickness. After adjustment for confounding factors, higher SUA levels were independently associated with plaque instability. In our study, SUA level was a possible surrogate marker of vulnerable plaques in nonculprit lesions of patients with ACS.

A consistent number of preclinical and clinical studies demonstrated that elevated SUA level is associated with CAD development [21–26]. Uric acid could contribute to CAD development through several mechanisms: vasoconstriction, inflammation, oxidative stress, and endothelial dysfunction. Recent studies show that uric acid stimulates chemokines, such as monocyte chemoattractant protein-1 (MCP-1), and inflammatory markers, such as high-sensitivity C-reactive protein, white blood cells, interleukin-1, interleukin-6, interleukin-10, interleukin-18, endothelin-1, and tumor necrosis factor-alpha, all contributing to CAD [27].

SUA level is significantly associated with the number of diseased vessels. SUA is an independent risk factor for multivessel disease by univariate analysis. High levels of SUA correlate with the severity of CAD in nondiabetic, nonhypertensive patients with ACS [28].

The overall risk of cardiovascular mortality has been shown to increase by 12% for each increase of 1 mg/dl in SUA level, and hyperuricemia increases the risk of death in women, as recently confirmed by a cross-sectional retrospective study of 607 premenopausal women [29]. The National Health and Nutrition Survey III (NHANES III) evaluated over 16,000 patients and concluded that SUA level in excess of 6 mg/dl is an independent risk factor for CAD [30, 31]. Kaya et al. demonstrated higher rates of in hospital and long-term major adverse cardiovascular events in patients with ST-segment elevation MI with high SUA level than in those with low SUA level [32]. Cardiovascular mortality, reinfarction, target vessel revascularization, and severe heart failure were observed more frequently in the high SUA group.

There is little information about the association between SUA level and coronary plaque characteristics [8, 33]. Elevated SUA level has been reported to be a predictor of poor coronary blood flow after primary PCI [34, 35]. No-reflow or slow-flow phenomenon occurs in patients with greater lipid content of coronary plaques [36, 37]. Using IB-IVUS, elevated SUA level has been associated with greater lipid content of coronary plaques in patients with ACS than in patients with normal levels, and increased SUA levels were associated with larger lipid content plaques in both genders [6, 7].

In clinical study, it is still unknown whether lowering SUA levels will provide stabilizing effect on coronary plaque. However, elevated SUA levels were associated with higher instability of plaque. So lowering SUA may be beneficial for secondary prevention in patients with acute coronary syndromes.

## 5. Limitations

There are some limitations to the present study. First, the number of patients was relatively small. Second, clinical information of long-term outcomes was not available, although elevated SUA level was suggested as an indicator of greater lipid content of coronary plaques. Third, we only analyzed plaques that were proximal to the culprit lesion. This made the studied segment shorter but the vessel size relatively uniform. Vessel size correlates with lipid content of the coronary plaque; this is why we evaluated nonculprit plaques at the segment proximal to the culprit lesion.

## 6. Conclusions

The present study showed that elevated SUA level is associated with greater lipid content of nonculprit coronary plaques in patients with ACS, and SUA level is a possible surrogate marker based on OCT.

## Figures and Tables

**Table 1:** Baseline characteristics.

Variable	Tertile			*p* value
	Low (*n* = 37)	Intermediate (*n* = 76)	High (*n* = 37)	
Men (%)	20 (54.05)^*∗*^	57 (75.00)	31 (83.78)	0.013
Age (years)	65.12 ± 10.03	65.24 ± 10.07	64.96 ± 9.98	0.77
hs-CRP (mg/L)	3.53 ± 0.31^*∗*^	6.46 ± 0.87^#^	12.17 ± 1.37^△^	<0.001
Hypertension	27 (72.97)	45 (59.21)	26 (70.27)	0.49
Diabetes mellitus	15 (40.54)	20 (26.31)	12 (32.43)	0.59
Dyslipidemia	23 (62.16)	53 (69.73)	26 (70.27)	0.68
Current smoker	14 (37.83)	31 (40.79)	15 (40.54)	0.95
STEMI	9 (24.32)	22 (28.95)	13 (35.14)	0.59
NSTEMI	7 (18.92)	13 (17.11)	8 (21.62)	0.85
Unstable angina	21 (56.76)	41 (53.95)	16 (43.24)	0.45
Cr (*μ*mol/L)	83.96 ± 19.34	85.66 ± 20.34	86.38 ± 22.15	0.78
Serum uric acid (*μ*mol/L)	223.65 ± 39.76^*∗*^	323.58 ± 33.37^#^	440.42 ± 59.93^△^	<0.001
LDL (mmol/L)	2.95 ± 0.60	2.89 ± 0.54	2.93 ± 0.59	0.41
HDL (mmol/L)	0.82 ± 0.13	0.78 ± 0.12	0.81 ± 0.14	0.57
TG (mmol/L)	1.41 ± 0.23	1.42 ± 0.25	1.40 ± 0.21	0.89
TC (mmol/L)	4.73 ± 1.10	4.78 ± 1.00	4.83 ± 1.18	0.92
Allopurinol	0	3	7	NA
Febuxostat	0	0	3	NA

^*∗*^Significance between low tertile group and intermediate tertile group; ^#^significance between intermediate tertile group and high tertile group; ^△^significance between low tertile group and high tertile group.

**Table 2:** Quantitative and qualitative optical coherence tomography findings on plaques stability.

Variable	Tertile			*p* value
	Low	Intermediate	High	
OCT quantitative data				
Minimum lumen area, mm^2^	6.02 ± 1.11^*∗*^	5.02 ± 1.46	5.38 ± 1.28^△^	<0.001
Fibrous-cap thickness, mm	0.15 ± 0.06^*∗*^	0.07 ± 0.01^#^	0.04 ± 0.01^△^	<0.001
Plaque length, mm	16.03 ± 8.37	15.61 ± 7.51	15.62 ± 7.09	0.915
Mean lipid arc (°)	169.41 ± 33.16^*∗*^	177.22 ± 37.76^#^	222.43 ± 47.65^△^	<0.001
Lipid length, mm	10.47 ± 7.27	11.10 ± 7.50	11.28 ± 7.12	0.608
OCT qualitative data, *n* (%)				
TCFA	19 (24.36)	43 (27.56)^#^	33 (42.30)^△^	0.028
Microvessel	21 (26.92)	51 (32.69)^#^	10 (12.82)^△^	0.005
Macrophage	17 (21.79)	46 (29.49)^#^	37 (47.43)^△^	0.002
Cholesterol crystal	23 (29.49)	49 (31.41)^#^	45 (57.69)^△^	<0.001

^*∗*^Significance between low tertile group and intermediate tertile group; ^#^significance between intermediate tertile group and high tertile group; ^△^significance between low tertile group and high tertile group.

**Table 3:** Correlations between OCT findings and serum uric acid.

OCT data	*r*	*p* value
Minimum lumen area	−0.11	0.04
Fibrous-cap thickness	−0.84	<0.001
Plaque length	0.009	0.87
Mean lipid arc	0.79	<0.001
Lipid length	0.06	0.28

**Table 4:** Univariate and multivariate logistic analyses for plaques stability.

	Univariate		Multivariate	
	OR (95% CI)	*p* value	OR (95% CI)	*p* value
Men	1.58 (0.94–2.67)	0.087	1.16 (0.65–2.06)	0.615
Smoker	0.80 (0.50–1.29)	0.36	0.72 (0.43–1.20)	0.206
Cr	0.99 (0.98–1.01)	0.933	1.00 (0.99–1.01)	0.963
LDL	1.25 (0.82–1.90)	0.307	1.32 (0.84–2.06)	0.227
Serum uric acid	1.10 (1.01–1.21)	<0.001	1.052 (1.029–1.076)	0.003
hs-CRP	1.20 (1.10–1.31)	<0.001	0.98 (0.83–1.14)	0.751
